# Promoting vocabulary learning during sleep at home using closed‐loop targeted memory reactivation

**DOI:** 10.1111/jsr.70000

**Published:** 2025-02-10

**Authors:** Federico Salfi, Aurora D'Atri, Benedetto Arnone, Domenico Corigliano, Giulia Amicucci, Lorenzo Viselli, Federica Naccarato, Fabiana Festucci, Daniela Tempesta, Michele Ferrara

**Affiliations:** ^1^ Department of Biotechnological and Applied Clinical Sciences University of L'Aquila L'Aquila Italy; ^2^ Department of Psychology Sapienza University of Rome Rome Italy

**Keywords:** closed‐loop, home setting, spindle activity, targeted memory reactivation, vocabulary learning, wearable EEG system

## Abstract

Targeted memory reactivation represents an established technique for promoting sleep‐dependent memory consolidation in laboratory studies. This investigation aimed to test the potentiality of a wearable electroencephalography‐based closed‐loop targeted memory reactivation system to boost vocabulary learning in home settings. In the evening, 24 adults (23.58 years ± 3.36 years, 19 females) were asked to learn the Italian translation of 40 pseudowords (test session). Subsequently, participants slept at their home wearing an electroencephalography headband (Dreem 2), and half of the pseudowords were acoustically re‐presented (cued) following the real‐time detection of slow waves. After the awakening, memory recall of the Italian translations was retested. The stimulation effect was evaluated by comparing the test–retest variations in memory accuracy between cued and uncued pseudowords. Moreover, we assessed the event‐related potentials and spectral perturbations induced by the cued stimuli during sleep, comparing the electrophysiological correlates of correctly translated pseudowords with incorrectly translated ones at the retest session. Closed‐loop targeted memory reactivation increased the translation accuracy for cued pseudowords (+8.6%), while no significant test–retest variation for uncued items was observed (−4.6%). Time–frequency analysis indicated a spectral power increase in the spindle frequency band coinciding with the second positive peak of the sound‐elicited slow wave as the correlate of successful morning recall. This study extended the targeted memory reactivation effectiveness in enhancing vocabulary learning to an ecological home environment, providing further support to the role of spindle activity in the targeted memory reactivation effect. A wearable closed‐loop targeted memory reactivation system could represent a memory‐enhancement tool in real‐world settings by promoting the hallmark sleep electroencephalographic rhythms of memory consolidation.

## 1 INTRODUCTION

Since its first experimental evidence in 1924 (Jenkins & Dallenbach, 1924), a century of research has firmly established the pivotal and active role of sleep in the process of memory consolidation (Brodt et al., 2023; Klinzing et al., 2019; Rasch & Born, 2013).

Current models suggest that the strengthening of memory traces is linked to the deepest stage of sleep, known as slow‐wave sleep (SWS; stage N3), when memory traces are spontaneously reactivated within hippocampal assemblies. This replay occurs together with other brain oscillations hallmarking SWS such as neocortical slow oscillations (SOs; < 1 Hz), thalamocortical spindles (11–16 Hz) and hippocampal ripples (80–140 Hz; Klinzing et al., 2019). The precise temporal coordination of these distinct electrophysiological patterns forms the neural basis for the gradual transformation of hippocampus‐dependent episodic memories into more stable and long‐lasting representations in the neocortex (Brodt et al., 2023; Klinzing et al., 2019).

In the last two decades, the understanding of the close relationship between sleep and memory has encouraged the development of neuromodulation techniques consisting of acoustic stimulations to non‐invasively boost memory consolidation during sleep (Cellini & Mednick, 2019).

Targeted memory reactivation (TMR) is a well‐established approach for promoting sleep‐dependent memory consolidation (Oudiette & Paller, 2013; Rasch et al., 2007). In TMR paradigms, specific learning episodes are designed so that a sensory cue (e.g. a sound) is matched with a target (e.g. a word) during wakefulness. Subsequently, the same sensory cues are re‐presented several times during specific sleep stages (typically N2 and/or N3) to promote the reactivation of memory traces and their consolidation (Hu et al., 2020). To date, several studies conducted in well‐controlled laboratory conditions confirmed the effectiveness of TMR in different memory domains, especially the declarative one (Carbone & Diekelmann, 2024; Hu et al., 2020).

A fascinating future direction of TMR consists of translating its application into real‐world settings. However, in most TMR studies the sensory stimuli were delivered during the night in an open‐loop manner (i.e. an experimenter started the stimulation during a predetermined sleep stage), preventing its application in a home environment.

In the last years, some studies tried to apply unsupervised TMR protocols in which auditory (Göldi & Rasch, 2019) or olfactory stimuli (Neumann et al., 2020; Vidal et al., 2022) were presented during the night without targeting specific sleep stages, offering promising but controversial results due to methodological limitations (lack of blindness about the experimental condition, potential cueing during wakefulness, awakenings by auditory presentation with paradoxical negative impact on memory).

To overcome these limitations, the optimization of algorithms that automatically identify specific sleep stages and brain rhythms in real‐time led to the ideation of closed‐loop TMR paradigms (CL‐TMR; Göldi et al., 2019; Ngo & Staresina, 2022; Shimizu et al., 2018). In CL‐TMR experiments, auditory cue presentation is controlled by an algorithm that analyses the ongoing sleep electroencephalography (EEG) activity and automatically delivers sounds during the depolarization state of the SOs. These laboratory studies showed a substantial stimulation‐dependent positive effect on declarative memory consolidation, and two of them (Göldi et al., 2019; Ngo & Staresina, 2022) provided experimental support for the assumption that SO up‐state may represent a privileged time window for TMR application.

In parallel, the technological progress of hardware solutions led to the development and commercialization of comfortable wearable devices to accurately monitor sleep physiological signals, with interesting implications for future studies in ecological settings (de Gans et al., 2024).

Reliable at‐home sleep EEG recording systems in combination with effective algorithms for automatized stimulus presentation during a target sleep stage opened the way to the application of EEG‐based but unsupervised TMR protocols outside laboratory boundaries (Borghese et al., 2022; Schwartz et al., 2022).

One example is represented by the Dreem 2 headband (DH; Rythm SAS, France), a 5‐electrodes wearable EEG system conceived to record, store, and automatically analyse ongoing sleep EEG data (Arnal et al., 2020). The headband is implemented with an algorithm to accurately detect in real‐time N3 sleep stage, and send brief sound stimuli in the ascending phase of slow waves (Debellemaniere et al., 2018).

In this CL‐TMR study, we exploited the DH features to deliver sounds associated with a prior learning episode after the real‐time detection of sleep slow waves. In particular, our CL‐TMR protocol aimed at boosting vocabulary learning while participants slept at their homes. We expected to confirm the well‐documented effect of TMR in supporting vocabulary acquisition (Göldi et al., 2019; Schreiner & Rasch, 2015). Additionally, we explored the possibility of using a wearable EEG device to investigate the underlying electrophysiological mechanisms, hypothesizing that the benefit of TMR could be related to increased spindle activity, as already reported by previous TMR studies (Antony et al., 2018; Göldi et al., 2019; Groch et al., 2017; Schreiner et al., 2015; Wang et al., 2019).

## 2 MATERIALS AND METHODS

### 2.1 Participants

We excluded people taking any medication, having a history of neurological/psychiatric disorders, and presenting poor sleep quality (Pittsburgh Sleep Quality Index ≤ 5; Curcio et al., 2013) or insomnia symptoms (Insomnia Severity Index ≤ 7; Castronovo et al., 2016). We recruited young Italian native speakers who did not share the bedroom with anyone, and who reported a habitual sleep duration of 7–9 hours and going to bed at 23:00–00:30.

A total of 31 participants were enrolled in the study. The final sample consisted of 24 subjects (mean age ± standard deviation: 23.58 ± 3.36 years; range: 19–30 years; 19 females), after the exclusion of seven participants due to technical problems with the instrumentation (*n* = 3), poor signal quality (*n* = 3), and difficulty to sleep with the DH (*n* = 1).

Participants gave written informed consent to participate in the study before the experiment. The study was approved by the Internal Review Board of the University of L'Aquila (n. 33, 29 June 2021), and was conducted according to the Declaration of Helsinki.

### 2.2 Experimental procedure

The entire data collection was performed outside the laboratory, in an ecological home setting.

The experiment included an adaptation night prior to the stimulation night. The morning before the adaptation night, participants came to the laboratory to receive the equipment for CL‐TMR and a laptop for the memory assessment. During the lab visit, subjects were instructed on the correct use of the overall instrumentation and the execution of the memory task.

During the adaptation night, participants slept at home wearing the DH, and the sleep EEG was recorded to provide them with feedback on the outcome of the first recording and offer advice to optimize the signal quality if necessary.

The baseline memory assessment (*test* session; see “*Memory assessment”* section for a comprehensive task description) was scheduled in the evening (at 18:30) preceding the CL‐TMR night.

During the subsequent night, participants slept with the CL‐TMR system, and the sound stimulations were applied (see the “*EEG acquisition and stimulation procedure”* section for details). Subjects were allowed to sleep according to their habits and wake up without an alarm. In the post‐stimulation morning, a second memory assessment (*retest* session) was scheduled 30 min after their final awakening.

### 2.3 Memory assessment

We employed a Word–Pseudoword Association Learning task (WPAL) in which participants were required to learn the Italian “translation” of a set of 40 acoustically presented pseudowords. We used word–pseudoword pairs, rather than semantically associated or unrelated real word pairs, to mitigate potential confounds due to semantic guessing and unintended word associations. Pseudowords were novel, pronounceable, non‐sense phoneme combinations compliant with phonotactic Italian rules that resemble real words in form but lack any pre‐existing semantic associations. This feature ensured that participants could not rely on prior knowledge or established language networks during the task. Instead, they must learn and remember the specific pairing between a real word and a pseudoword, which allowed us to more accurately assess associative learning and the CL‐TMR effect on memory consolidation. The full list of word–pseudoword pairs used in this study is reported in Table S1 of the Supplementary material.

During the task execution, participants were asked to be alone in a quiet room with the door closed and activate the “airplane mode” on their smartphones to minimize potential sources of distraction.

The WPAL consisted of three phases: *encoding*, *training* (three trials), and *test*. Each trial was separated by a 2‐min break. During the *encoding*, pseudowords were presented in randomized order via loudspeakers, followed by the visual presentation of their Italian translation (3000 ms) on the PC monitor. Subjects were requested to memorize as many translations as possible.

In the three *training* trials, pseudowords were randomly presented again followed by a question mark, and participants had to type the correct translation within a maximum of 10 s. Afterward, feedback about the correctness of the response appeared on the monitor (correct answer: a green check mark; wrong answer: a red X mark) followed by the exact translation. In the *test* phase, the translation of all the pseudowords was again requested, this time without any response feedback. To avoid floor/ceiling effects, a pilot study was performed on 28 young adults (24.78 years ± 2.86 years; range: 22–30 years; 18 females) showing medium task difficulty (mean correct responses at the *test* phase ± standard deviation: 21.89 ± 5.58).

Pseudoword sounds were produced using the Read Aloud feature of Microsoft Word (*Luca*'s voice) and recorded with the Audacity software (version 3.1.3). Sound lengths were adapted to reduce duration heterogeneity (mean length ± standard deviation: 580 ± 67 ms).

The task has been programmed in Superlab 5.0.5 (Cedrus, San Pedro, California, USA) and remotely performed using the same software on the laptop that subjects received during the lab visit.

### 2.4 EEG acquisition and stimulation procedure

Brain cortical activity was recorded using the DH via three frontal and two occipital dry electrodes, resulting in seven bipolar signals (Fp1‐O1, Fp1‐O2, Fp1‐F7, F8‐F7, F7‐O1, F8‐O2, Fp1‐F8; sampling rate: 250 Hz). A recent validation study (Arnal et al., 2020) showed that the device algorithm can provide automatic sleep staging with an accuracy close to the consensus of five expert sleep scorers. The headband is also implemented with another algorithm for the real‐time detection of N3 sleep stage to deliver brief sounds via a bone conduction system (pink noises of 100 ms) in the ascending phase of SOs, with a high degree of phase‐precision (mean ± standard deviation: 45° ± 52° for a target phase angle of 45°, where 90° corresponds to the slow‐wave positive peak; Debellemaniere et al., 2018).

In our study, CL‐TMR was managed by a self‐developed sound‐delivering console (SDC) connected to the DH via a 3.5‐mm audio jack output. SDC consisted of a small box that was placed near the bed (i.e. on the nightstand) containing an Arduino‐uno‐based hardware. During the detection of a SO up‐state, the DH generated a pink noise signal that was redirected from bone conduction to the external jack output and served solely as a trigger for the SDC. The SDC, upon receiving and amplifying this signal, managed the presentation of pseudowords through built‐in loudspeakers (45 dB) with a fixed delay of 750 ms. Thus, only the the pseudoword delivered by the SDC was audible to participants in the stimulation condition. Although this approach implies that the stimulation is not phase‐locked but rather occurs after the emergence of a SO, the delay was specifically chosen to minimize a systematic sound presentation during the hyperpolarization state of slow waves due to the inherent SDC latency and pseudoword duration. This choice was informed by recent CL‐TMR investigations (Göldi et al., 2019; Ngo & Staresina, 2022) that compared the effects of stimulation during the up‐state and down‐state, proposing the depolarization (up) state as the optimal phase for promoting memory consolidation. Detailed analyses evaluating the phase of stimulation across participants were provided in the Supplementary material. The SDC was powered by rechargeable cells, and it included a microSD connector so that the sounds to be presented would be selected from a microSD drive.

According to the sound presentation algorithm of DH, the stimulation began after 5 min of stable N3 with a minimum stimulation interval of 9 s. If a movement or alpha rhythm after stimulation was detected, stimulation was interrupted for 30 s.

Similar to other TMR studies on vocabulary learning (Göldi & Rasch, 2019; Schreiner & Rasch, 2015), nocturnal stimulation consisted of presenting half of the pseudowords (cued) included in the WPAL task. The cued pseudowords included half of those ones participants correctly translated at the baseline *test* session, and half of those ones they did not translate correctly. The pseudowords were individually and randomly selected for each participant using an automatic algorithm written in MATLAB (version 2018a). To ensure that the selection of pseudowords for TMR was not biased by translation difficulty potentially linked to sound symbolism (Züst et al., 2019), we calculated the baseline recall rates across participants for each pseudoword and compared the mean difficulty between cued and uncued ones. No significant difference was found (*p* = 0.671), indicating that the random selection of pseudowords for TMR was not biased towards easier or harder ones (see “Translation difficulty and selection of TMR stimuli” section of Supplementary material). Concurrently, the DH stimulation algorithm included the random presentation of sham stimuli, where slow waves were targeted but no sound was delivered.

Participants received a total of 167.00 ± 61.59 (mean ± standard deviation) CL‐TMR stimuli (~8 repetitions for each cued pseudoword) and 56.25 ± 21.41 sham stimulations. Information and sleep metrics about CL‐TMR nights retrieved from the DH output are reported in Table S2 of the Supplementary material.

### 2.5 Sleep EEG data

#### 2.5.1 Preprocessing

The EEG data were preprocessed using EEGLAB (version 2023.1; Delorme & Makeig, 2004). The data accessible for analysis were already filtered in the 0.4–18‐Hz range, reflecting the default bandpass filter settings applied by the DH system. Subsequently, data were segmented into trials of 14 s, centred on the stimulus/sham onset, for further analysis.

Signal quality and presence of artefacts were evaluated by visually inspecting all trials focusing on frontal‐occipital derivations (F7‐O1, F8–O2, Fp1‐O1, Fp1‐O2), leading to the exclusion of Fp1‐O1 and Fp1‐O2 derivations from further analyses due to the higher rate of poor signal quality to optimize the tradeoff between the number of analysable epochs and channels. Subsequently, artefact rejection was manually performed focusing on F7‐O1 and F8–O2 derivation.

Epochs were first categorized into sham and CL‐TMR trials. CL‐TMR trials were further classified into “Remembered” and “Not remembered” items on the basis of the behavioural performance at *retest*. The “Remembered” category referred to pseudowords delivered during sleep whose translation was retrieved at *retest*. “Not remembered” items included those pseudowords for which no correct translation was provided by participants at the morning *retest*.

#### 2.5.2 Event‐related potential (ERP) and event‐related spectral perturbation (ERSP) computation

Brain response evoked by sound stimulations was evaluated by calculating ERPs using EEGLAB (Delorme & Makeig, 2004). ERPs were obtained by averaging trials within each category (CL‐TMR, Sham, Remembered, Not remembered) for each participant. Afterward, data were baseline‐corrected to the pre‐cue segment from −4500 to −3500 ms to avoid the inclusion of the SO used to trigger the stimulations in the baseline window. The resulting ERPs were averaged between F7‐O1 and F8‐O2 derivations.

Time–frequency representation was computed using the “*newtimef*” EEGLAB function to evaluate the spectral correlates of CL‐TMR. Each epoch was convolved with a complex Morlet wavelet for frequencies ranging from 2 to 18 Hz, with 0.1‐Hz frequency resolution and 4‐ms time steps. We used an adaptive number of wavelet cycles that started from 3 at the lowest frequency (2 Hz) and linearly increased until 13.5 cycles at the highest frequency (18 Hz). This approach has been chosen to optimize the tradeoff between temporal and frequency resolution across the analysed spectrum (Delorme & Makeig, 2004). ERSP was obtained by dividing spectral power values at each time–frequency point by the mean spectral power in a pre‐stimulus time window (from −4500 to −3500 ms) at the same frequency bin, and averaging the power ratio values across trials of the same category (Remembered, Not remembered) for each participant. Finally, mean ERSPs were converted in decibels [dB = 10*log_10_(power ratio)] and averaged between F7‐O1 and F8‐O2 derivations.

### 2.6 Statistical analysis

#### 2.6.1 Behavioural data

To investigate the impact of CL‐TMR on translation recall, we conducted a logistic generalized linear mixed model (GLMM) analysis on R software using the “*GAMLj*” (version 3.0.0) module. Data were analysed at the single response level without aggregation (total number of observations = 1920), allowing us to take the full response patterns into account without averaging over individual trial‐by‐trial responses. The model included the participants' responses (correct, incorrect) as the dependent variable. Fixed effects in the model included *stimulus type* (cued, uncued), *session* (test, retest), and their interaction. The model also included random intercepts for *pseudoword* and *subject* to account for the repeated measure nature of data.

The logistic mixed effect model specified was:

$$\begin{array}{l} \text{Response}∼1+\text{stimulus type}+\text{session}+\text{stimulus type}:\text{session}\\ \;+\;\left(1|\text{pseudoword}\right)+\left(1|\text{subject}\right). \end{array}$$

Four planned comparisons with Bonferroni correction were performed in case of significant interaction effects using the “*emmeans*” R package (Lenth et al., 2022). We tested potential differences between sessions for each stimulus type (i.e. cued‐test versus cued‐retest, uncued‐test versus uncued‐retest). Moreover, we were also interested in comparing memory performance for cued and uncued pseudowords at each time point to investigate possible baseline or recall differences (i.e. cued‐test versus uncued‐test, cued‐retest versus uncued‐retest). All tests were two‐tailed, and a *p* < 0.05 was considered significant.

#### 2.6.2 Sleep EEG data

The ERPs of CL‐TMR and Sham stimuli were compared to evaluate the brain response evoked by sound stimulation during sleep. Moreover, a comparison between ERPs of Remembered and Not remembered words was performed to address potential differences in sound‐elicited cortical activity that characterized the subsequent memory recall. Finally, similar to previous TMR investigations on vocabulary learning (Cordi et al., 2018; Göldi et al., 2019; Schreiner & Rasch, 2015; Wick & Rasch, 2023; Wilhelm et al., 2020), the spectral correlate of sound presentation associated with successful memory retrieval at *retest* was evaluated by comparing the ERSP of Remembered and Not remembered stimuli.

Statistical analysis of ERP and ERSP data was performed using the FieldTrip toolbox (Oostenveld et al., 2011). All the analyses described in this paragraph consist of a paired samples *t*‐test with a cluster‐based correction for multiple comparisons. The correction was implemented using a Monte Carlo method with 1000 random permutations (Manly, 1997). The significance threshold for the cluster formation in the time domain (ERP) and time–frequency domain (ERSP) was set at 0.05, and the overall alpha level was also 0.05 (all two‐tailed). In the case of significant time–frequency clusters, paired sample *t*‐tests comparing Remembered and Not remembered stimuli on the mean power variation within the clusters were performed.

## 3 RESULTS

### 3.1 CL‐TMR effect on vocabulary memory

**FIGURE 1:**
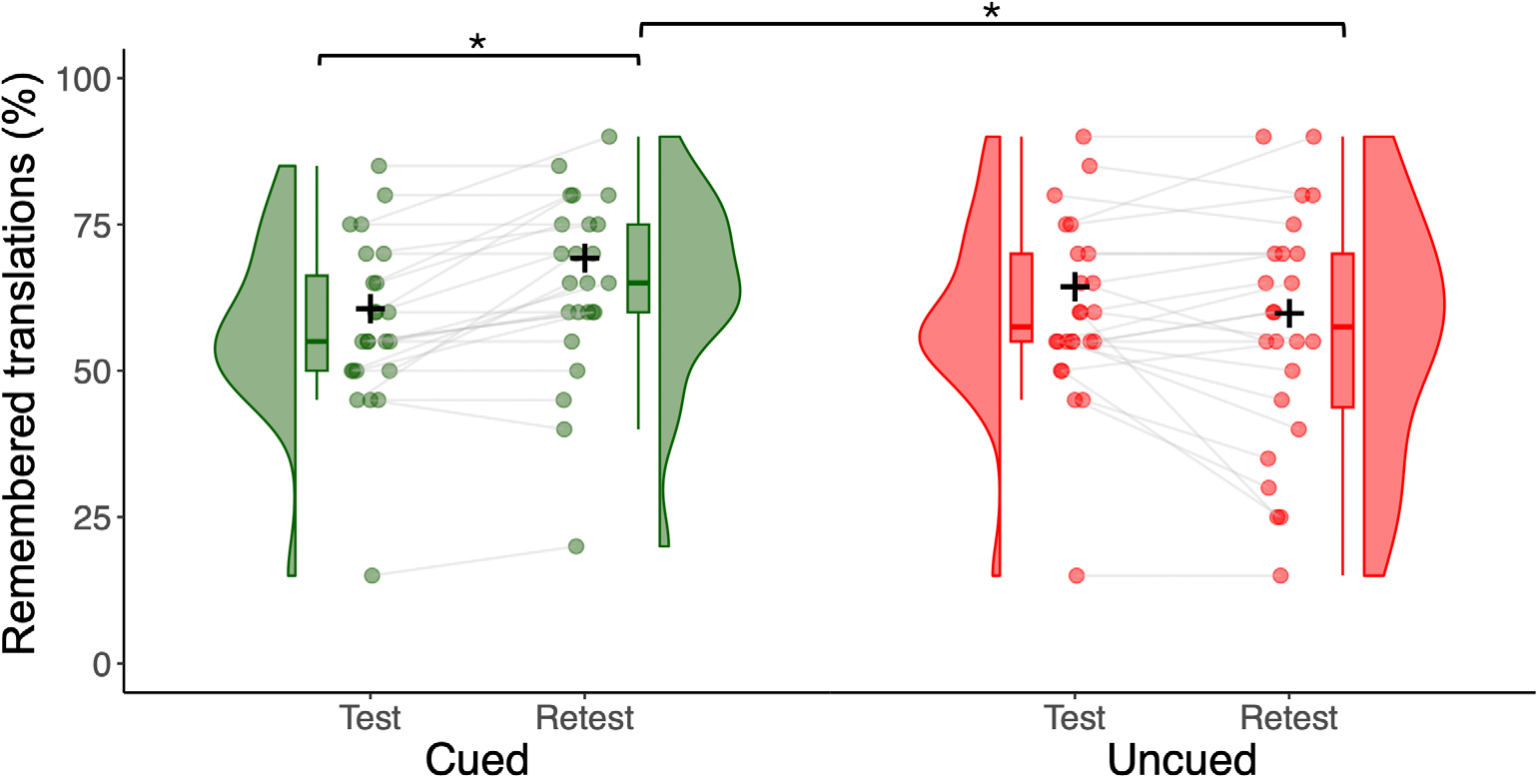
Remembered translations (%) at *test* and *retest* sessions for cued (green) and uncued (red) stimuli, and results from Bonferroni‐corrected planned comparisons. Raincloud plots combine a density plot, a box plot, and a jitter plot for each stimulus type (cued, uncued) and session (test, retest). Plus symbols represent estimated marginal means from the logistic GLMM analysis, and asterisks indicate significant differences (*p < 0.050, corrected). GLMM, generalized linear mixed model.

Logistic GLMM showed a non‐significant effect of the *session* [*exp(B)* = 1.098, *95% CI* = 0.891–1.353, *p* = 0.381] and *stimulus type* factors [*exp(B)* = 1.139, *95% CI* = 0.916–1.415, *p* = 0.242]. Meanwhile, the model indicated a significant *session* × *stimulus type* interaction [*exp(B)* = 1.781, *95% CI* = 1.172–2.706, *p* = 0.007]. Planned comparisons (Figure 1) showed a significant increase in the number of correctly translated pseudowords at *retest* compared with *test* session (+8.6%, *p*_corrected_ = 0.048) for the cued stimuli. On the other hand, no *test–retest* significant variation emerged for the uncued stimuli (**−**4.6%, *p*_corrected_ = 0.780). As expected, no difference was found at *test* between cued and uncued stimuli (**−**3.7%, *p*_corrected_ = 1.000). Finally, cued stimuli were associated with a higher recall rate of the correct translations at *retest* than uncued ones (+9.5%, *p*_corrected_ = 0.028). The same pattern of results was confirmed after removing one outlier at the *test* session from the analyses.

### 3.2 ERPs of stimulations

**FIGURE 2:**
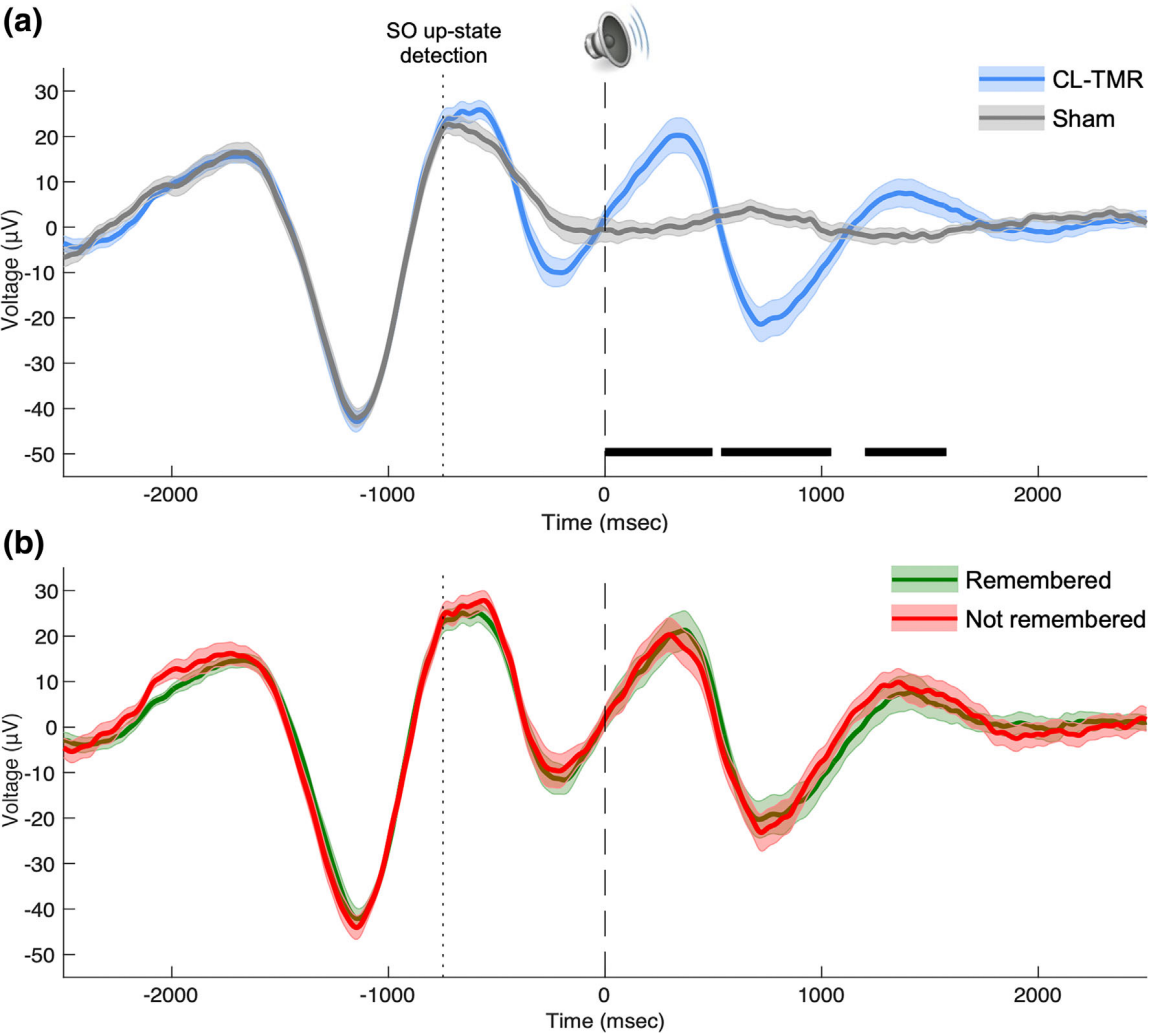
(a) Grand‐average ERPs of CL‐TMR (blue line) versus Sham stimulation (grey line), and (b) of cued pseudowords whose translation was remembered (green line) versus not remembered (red line) at *retest* across all participants and channel derivations (F7‐O1 and F8‐O2). The timing of sound presentation is indicated by the dashed line. The dotted line represents the time point of detected SO up‐state by the Dreem 2 algorithm. Shaded area represents the standard error of the ERPs across participants. Horizontal lines indicate significant differences from cluster‐based permutation tests (*p* < 0.050, corrected). CL‐TMR, closed‐loop targeted memory reactivation; ERP, event‐related potential; SO, slow oscillation.

On average, the onset of sounds occurred in the ascending phase of the slow wave (Figure 2). The ERP comparison between CL‐TMR and Sham stimuli showed a clear slow wave induction after sound onset. Specifically, the analysis indicated that the sound presentation induced a first positive peak at ~340 ms, a subsequent negative peak at ~710 ms, and a second positive peak at ~1360 ms compared with Sham (all *p*_corrected_ < 0.050; Figure 2a).

No difference emerged between the amplitude of the brain response evoked by cued pseudowords subsequently remembered at *retest* and those not remembered (Figure 2b).

### 3.3 Spectral correlates of successful CL‐TMR

**FIGURE 3:**
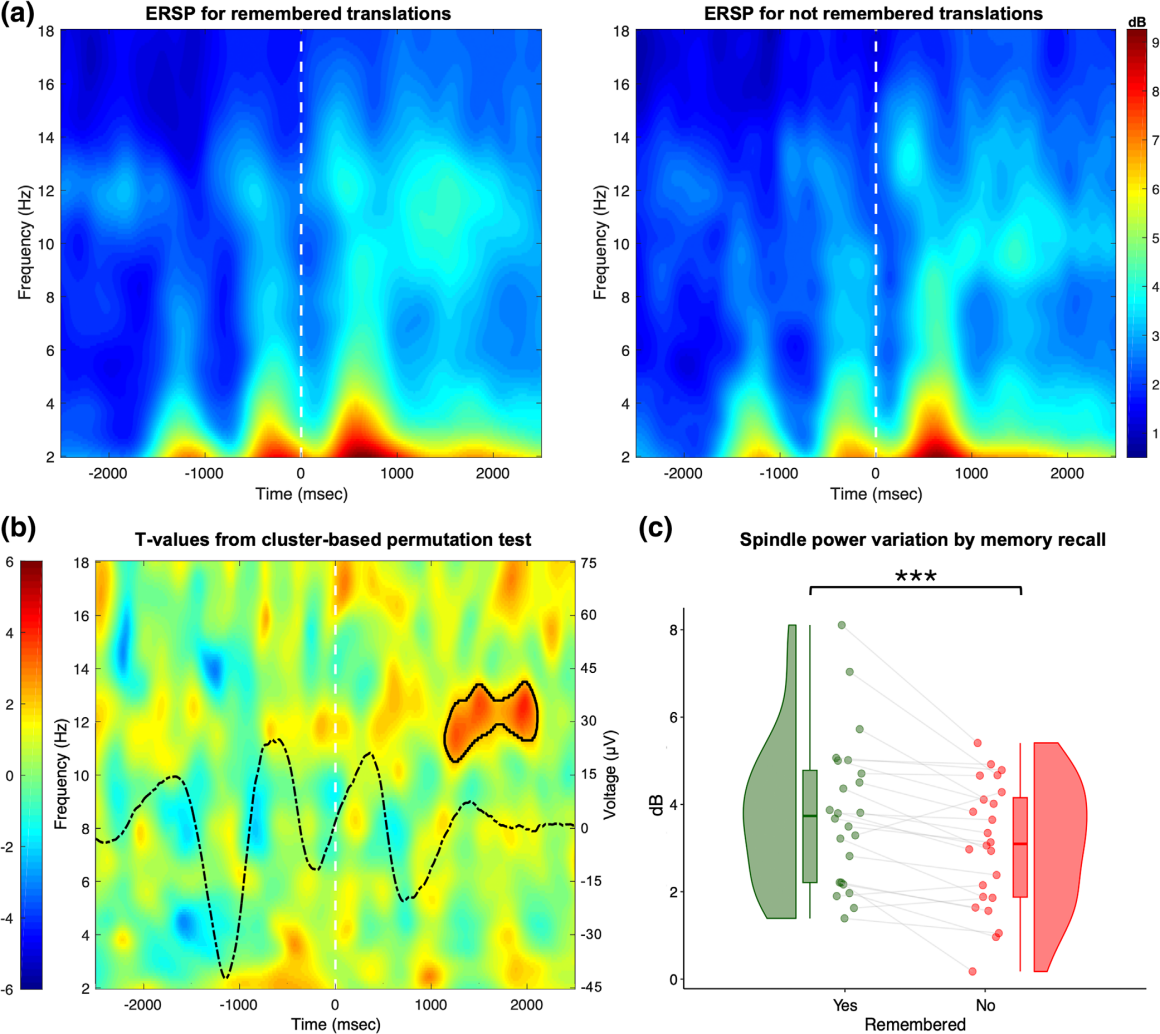
(a) ERSP following time–frequency decomposition for cued pseudowords whose translation was remembered (left) and not remembered at *retest* (right). The figures depict the mean values between F7‐O1 and F8‐O2 derivations. The timing of sound onset is indicated by the dashed line. (b) *t*‐values obtained from the Monte Carlo permutation test. Black contour indicates the time–frequency cluster showing a significant difference (*p* < 0.050, corrected). The superimposed dashed line represents the ERP of pseudowords correctly translated at the *retest* session (voltage values are indicated in right *y*‐axis). (c) Raincloud plot of spectral power variation within the significant cluster shown in (b) for the remembered (green) and not remembered (red) translations at *retest*. Asterisks indicate a significant difference (***p < 0.001). ERP, event‐related potential; ERSP, event‐related spectral perturbation.

The ERSP comparison between cued pseudowords correctly translated at *retest* and those not correctly translated (Figure 3a) indicated a significant time–frequency cluster in the spindle range (10.5–13.5 Hz) at 1140–2100 ms after sound onset (*p*_corrected_ < 0.050; Figure 3b). Stronger stimulus‐induced spindle power variation within the time–frequency cluster characterized successful memory recall at the morning *retest* (*t* = 4.165, *p* < 0.001; Figure 3c).

## 4 DISCUSSION

This study aimed to evaluate the potentiality of an EEG‐based CL‐TMR protocol in which sound stimulation was guided by a wearable headband in an ecological home setting. In particular, we tested the feasibility and effectiveness of our approach in promoting declarative memory consolidation, providing evidence for a CL‐TMR‐induced benefit in the acquisition of a novel language in a real‐world setting.

After studying the Italian translation of a set of pseudowords, participants slept in their homes while part of the pseudoword list was delivered upon the detection of slow waves. At the awakening, subjects showed an increased accuracy (+8.6%) in translating those items that had been re‐presented during sleep, while no significant test–retest differences marked the translation performance of uncued pseudowords (**−**4.6%). Consequently, participants' ability to translate pseudowords was higher for stimuli delivered during the night than those that had not been re‐presented (+9.5%).

Our findings align with an extensive body of laboratory studies demonstrating that TMR during non‐rapid eye movement (NREM) sleep can facilitate the consolidation of declarative memories (for meta‐analysis, see Hu et al., 2020). Moreover, this study confirmed the effectiveness of TMR in promoting foreign vocabulary acquisition, as shown by previous investigations in which participants learned new Dutch words and their German translations (for review, see Schreiner & Rasch, 2017).

Two recent studies performed in home settings already employed the DH to apply CL‐TMR (Borghese et al., 2022; Schwartz et al., 2022). However, based on the aims of these experiments (modulating social anxiety or treating patients with nightmare disorder), sound stimulations were presented during rapid eye movement (REM) sleep. On the other hand, this is the first study that exploited a wearable EEG device to replicate the well‐documented TMR effects on declarative memory outside the laboratory boundaries while exploring the underlying electrophysiological mechanisms. Our findings complement those of a recent preliminary investigation addressing the use of TMR in a portable and educational setting (Mar'i et al., 2024), supporting the idea that auditory stimulation can be successfully applied with unsupervised, real‐time detection of brain rhythms.

The potentiality of TMR as a declarative memory enhancement tool in real‐world settings has been already shown by a recent study that developed a portable system using movement and heart‐rate data to identify sleep stages and deliver sounds by a smartphone (Whitmore et al., 2022). This study showed that TMR reliably improved spatial memory, consistent with effects observed in laboratory experiments.

At the electrophysiological level, pseudowords re‐presented during sleep and correctly translated upon awakening elicited a similar cortical response compared with those not correctly recalled. A previous investigation on the effects of TMR on vocabulary learning (Schreiner & Rasch, 2015) suggested that analysing ERP responses separately for specific memory categories, such as Gain (i.e. words not remembered pre‐sleep but recalled post‐sleep) and Loss (i.e. words remembered pre‐sleep but forgotten post‐sleep), revealed distinct ERP patterns. In our study, we were unable to replicate this analytical approach due to the low representation of Gain and Loss trials in our dataset. As a result, our analyses focused on the broader categories of Remembered and Not Remembered, potentially explaining the absence of a subsequent memory effect in the ERP results, as well as preventing us from providing a more detailed overview of the memory processes affected by TMR based on pre‐sleep performance. However, the time–frequency analysis showed that the cueing of the subsequently recalled translations induced a higher spindle activity (10.5–13.5 Hz) coinciding with the second positive peak of the sound‐evoked slow wave. This spindle activity variation could be therefore interpreted as the correlate of the effective reactivation of the associated memory trace and a predictor of the subsequent successful memory retrieval during the morning retest session. This finding is in line with the initial hypothesis, as the association between increased spindle activity and the memory enhancement effect of TMR has been already reported by previous investigations adopting both auditory (Antony et al., 2018; Göldi et al., 2019; Groch et al., 2017; Schreiner et al., 2015; Wang et al., 2019) and olfactory stimuli (Cox et al., 2014; Rihm et al., 2014).

In this study, auditory cues were delivered with a fixed interval (750 ms) after detecting the up‐state of slow waves. This stimulation procedure aimed to minimize the possibility of a systematic sound presentation during the hyperpolarizing phase, based on a growing body of TMR literature proposing the SOs down‐states as the suboptimal time window to promote memory consolidation (Göldi et al., 2019; Ngo & Staresina, 2022). On average, our approach led the sounds to be presented during the ascending phase of slow waves (Figure 2). However, we acknowledge that it does not represent an ideal system to systematically target the SO up‐state. Indeed, the process of detecting a slow wave and subsequently delivering the stimulation after such a long and fixed interval introduces variability in the oscillatory activity following the detected wave. This consideration explains the variability in the stimulated phase across and within our participants (see “*Phase of stimulation*” section of Supplementary material). Different algorithms may be adopted (for overview, see Esfahani et al., 2023) to further optimize the behavioural and electrophysiological outcomes of TMR when applied in home settings.

Several limitations of the study should be disclosed. First, the sample size was relatively small and included a group of young healthy individuals, reducing the generalizability of the results to populations of different ages or clinical groups. Additionally, although the DH headband accurately detects sleep stages (Arnal et al., 2020; Debellemaniere et al., 2018), the use of a self‐applied wearable device in a home environment introduces potential variability in EEG signal quality. This variability is particularly pronounced with dry electrodes, which lack impedance checks and are prone to instability during unsupervised recordings. Despite extensive efforts to optimize signal acquisition, including iterative preprocessing and careful artefact rejection, data loss due to poor signal quality affected several participants and required exclusion of certain derivations. Moreover, the referencing scheme of the DH combined with the default bandpass filtering limited the scope of our EEG analyses. While these constraints significantly reduced the generalizability of our analyses to laboratory investigations relying on traditional polysomnographic amplifiers, our findings support the possibility of using wearable EEG technology for exploratory analyses on the effects of TMR in real‐world, unsupervised settings.

Finally, the DH was well tolerated, with only one participant withdrawing from the study due to difficulties in sleeping with the headband. However, the potential of using the DH device for TMR might end with this study. Indeed, due to changes in company policies, the stimulation system was discontinued, preventing the use of DH for future neuromodulation studies. Fortunately, several alternative wearable EEG sleep trackers have been commercialized in recent years (de Gans et al., 2024), with some of them specifically designed to apply closed‐loop auditory stimulation (Zeller et al., 2023).

## 5 CONCLUSION AND FUTURE DIRECTIONS

The present study highlights the feasibility of using portable, self‐applied, wearable EEG devices for closed‐loop neuromodulation in real‐world environments. This evidence opens up promising avenues for future applications of TMR not only in healthy individuals but also in clinical populations where sleep‐related cognitive impairments are prevalent (e.g. mild cognitive impairment, Alzheimer's disease). The possibility of implementing TMR outside the lab could extend memory‐enhancing interventions to a broader range of individuals, improving accessibility and adherence to sleep‐based treatments and paving the way to long‐term interventions. Refining the technology and algorithms to further improve the precision and reliability of sound delivery would enhance the efficacy of home‐based TMR protocols.

In summary, this study demonstrated the potential of TMR to enhance declarative memory consolidation in a home setting using wearable EEG devices. Our findings provided further evidence for the role of sleep in memory consolidation and confirmed the importance of spindle activity in this process. As the field moves towards more personalized and accessible neuromodulation interventions, home‐based TMR protocols could play a pivotal role in future cognitive enhancement and rehabilitation strategies.

## AUTHOR CONTRIBUTIONS

**Federico Salfi:** Data curation; conceptualization; software; visualization; methodology; investigation; formal analysis; funding acquisition; writing – original draft; writing – review and editing. **Aurora D'Atri:** Formal analysis; funding acquisition; writing – review and editing. **Benedetto Arnone:** Methodology; software; writing – review and editing. **Domenico Corigliano:** Investigation; writing – review and editing. **Giulia Amicucci:** Writing – review and editing. **Lorenzo Viselli:** Writing – review and editing. **Federica Naccarato:** Writing – review and editing. **Fabiana Festucci:** Writing – review and editing. **Daniela Tempesta:** Writing – review and editing. **Michele Ferrara:** Conceptualization; methodology; investigation; supervision; funding acquisition; writing – review and editing.

## CONFLICT OF INTEREST STATEMENT

The authors declare no conflicts of interest.

## Supporting information


**DATA S1** Supporting Information.

## Data Availability

The data that support the findings of this study are available from the corresponding author upon reasonable request.
