# Supplementary material for: Promoting vocabulary learning during sleep at home using closed‐loop targeted memory reactivation
Source: J Sleep Res. 2025 Feb 10;34(6):e70000. doi: 10.1111/jsr.70000 (PMC12592824; doi:10.1111/jsr.70000)
Supplement: Supplementary file 1 — DATA S1 Supporting Information. [file JSR-34-e70000-s001.pdf]

Promoting vocabulary learning during sleep at home using closed-loop targeted memory reactivation

Federico Salfi[1], Aurora D'Atri[1]*, Benedetto Arnone[1], Domenico Corigliano[1,2], Giulia Amicucci[1], Lorenzo Viselli[1], Federica Naccarato[1], Fabiana Festucci[1], Daniela Tempesta[1], Michele Ferrara[1]

[1]Department of Biotechnological and Applied Clinical Sciences, University of L'Aquila, L'Aquila, Italy
[2]Department of Psychology, Sapienza University of Rome, Rome, Italy

*Corresponding author
Prof. Aurora D'Atri, Ph.D.
Department of Biotechnological and Applied Clinical Sciences
University of L'Aquila
Via Vetoio
67100 L'Aquila (AQ)
Italy
aurora.datri@univaq.it

Supplementary material

Table S1. List of pseudowords and the respective Italian word used in the memory task. The English translation of each Italian word is also provided.

Pseudowords	Italian words	English words
tagnidro	affetto	affection
selughi	bandiera	flag
chigo	bellezza	beauty
vagliobo	chiave	key
pretole	commedia	comedy
quicra	comodino	nightstand
fispumo	disastro	disaster
gnufo	dottore	doctor
odema	finestra	window
gotera	fischio	whistle
padiga	giacca	jacket
teschipo	giardino	garden
linofo	gloria	glory
glusco	gomito	elbow
mocrebo	gusto	taste
presema	hotel	hotel
vranoma	identità	identity
tucca	inferno	hell
sceto	insulto	insult
vulno	matita	pencil
lotrafe	metallo	metal
borma	metodo	method
lusbri	miele	honey
toripla	moneta	coin
sbiplo	orrore	horror
tazio	padella	pan
tacipaca	pistola	gun
cilosabe	poltrona	armchair
molpa	scarpa	shoe
rasello	sorriso	smile
primosta	specchio	mirror
docaro	spiaggia	beach
obimone	spina	thorn
bileggi	statua	statue
sefone	tappeto	carpet
dunibli	teoria	theory
pemuscla	treno	train
riffri	uccello	bird
lonaccio	ulivo	olive tree
bitto	viale	avenue

Table S2. Information and sleep metrics of CL-TMR nights (n=24) retrieved from the Dreem 2 output. The definitions were obtained from the EEG headband manual.

Variable	Mean ± standard deviation	Definition
Start time (hh:mm ± min)	00:18 ± 65.08	Record start time with headband on head
Sleep onset time (hh:mm ± min)	00:32 ± 60.60	Beginning of the night (beginning of the falling asleep period):<br>First stable switch from open eyes to close eyes. By stable we mean that subject stays at least 2 min with closed eyes.
Sleep start time (hh:mm ± min)	00:44 ± 65.86	Moment of the first sleep period of the user (end of the sleep onset period):<br>First stable sleeping epoch, at least 5 minutes of consecutive sleeping.
Sleep stop time (hh:mm ± min)	08:40 ± 80.61	Sleep stop time: Time of the last wake up
Stop time (hh:mm ± min)	08:42 ± 80.34	End of the record: when the user stops the record or when he last removes the headband from his head.
Duration (min)	503.78 ± 70.52	Record duration: stop time–start time
Sleep duration (min)	464.67 ± 69.61	Duration of sleep stages (on smoothed hypnogram)
Sleep onset duration (min)	21.37 ± 12.35	Sleep onset duration: sleep start time– sleep onset time
Number wakes	2.54 ± 2.43	Number of wakes (between sleep start time and sleep stop time)
WASO (min)	11.71 ± 14.58	Wake after sleep onset duration (between sleep start time and sleep stop time)
Duration wake (min)	34.29 ± 21.21	Duration of wake stage
Duration N1 (min)	2.94 ± 3.89	Duration of N1 sleep stage
Duration N2 (min)	224.48 ± 58.26	Duration of N2 sleep stage
Duration N3 (min)	107.77 ± 30.18	Duration of N3 sleep stage
Duration REM (min)	134.69 ± 32.65	Duration of REM sleep stage

Translation difficulty and selection of TMR stimuli

To evaluate whether the pseudowords selected for TMR (cued) differed in difficulty at baseline compared to the uncued ones, we performed a control analysis. Specifically, we calculated a learning difficulty score for each pseudoword, defined as the percentage of participants who had translated it correctly during the evening test session. This score provides an objective measure of how challenging each pseudoword was for participants at baseline. We then conducted a Linear Mixed Model analysis using the *"GAMLj"* package in R. The model included the difficulty score as the dependent variable, the stimulation condition (cued vs. uncued) as a 2-level fixed effect, and a random intercept for participants. A total of 960 observations were analyzed.

The results revealed no significant difference in baseline difficulty scores between cued (mean ± standard deviation: 58.7% ± 2.7%) and uncued pseudowords (57.3% ± 2.7%; B=0.006, 95% CI =-0.023–0.035, p=0.671). These findings indicate that the random selection of items for TMR was unbiased with respect to their difficulty level, excluding a potential confounding factor related to baseline translation performance.

Phase of stimulation

To evaluate the slow wave phase during which stimulation occurred, the signal of each trial was averaged across the available derivations (F7-O1 and F8-O2). The resulting signal, which had already undergone high-pass filtering at 0.4 Hz (as per the default Dreem 2 preprocessing), was further low-pass filtered at 1.5 Hz using a zero-phase finite impulse response filter to minimize phase distortion. Subsequently, the Hilbert transform was applied to the signal to extract the instantaneous phase at the time of each stimulation. The mean stimulation phase was calculated for each participant and tested for non-uniformity using the Rayleigh test. The same test was applied at the single-trial level within each subject to evaluate whether the phases of stimulation were non-uniformly distributed. The grand mean stimulation phase (± standard deviation) across participants was 30.56° ± 54.18° (where 90° corresponds to the slow oscillation positive peak) and the Rayleigh test at the group level revealed significant non-uniformity in the phase distribution (p<0.001), confirming a preference for stimulation during the ascending phase of the slow waves (Figure S1). At the individual level, significant phase clustering was observed in 14 out of 24 participants (p<0.05). Among these, only one participant received stimulations preferentially during the down-state (227.29°), while all others were within the 0°–180° range (36.64° ± 24.67).

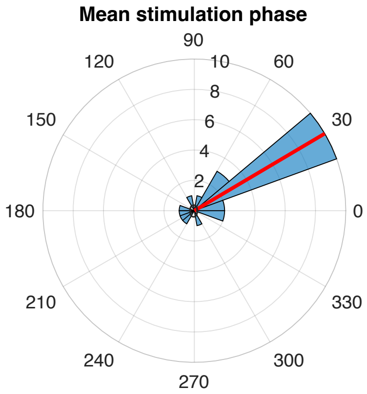

Figure S1. Phase distribution of stimulation across participants. The rose plot represents the mean stimulation phase for each participant, calculated from individual trials. The length of each 20° bin indicates the number of participants with stimulation phases falling within that angular range. The red line indicates the grand mean phase across participants.
